# Septal-to-basal ventricular peak activation time determined by vectorcardiography as a potential new pre-screening parameter for preclinical dilated cardiomyopathy in Doberman Pinschers

**DOI:** 10.3389/fvets.2025.1582006

**Published:** 2025-04-07

**Authors:** Margot Gheeraert, Gerhard Wess, Gitte Mampaey, Jenny Eberhard, Peter Gheeraert, Jan De Pooter, Luc Duchateau, Pascale Smets

**Affiliations:** ^1^Faculty of Veterinary Medicine, University of Ghent, Ghent, Belgium; ^2^Ludwig-Maximilians-University of Munich, Munich, Germany; ^3^Faculty of Medicine and Health Science, University of Ghent, Munich, Germany

**Keywords:** ECG, VCG, canine, vector, RELF

## Abstract

**Background:**

Early diagnosis of preclinical dilated cardiomyopathy (DCM) remains challenging in primary veterinary medicine due to the need for echocardiography and 24-h Holter electrocardiogram (ECG) recording. A readily available pre-screening tool to identify dogs at high risk could optimize current screening practice. Electrocardiographic methods have not been investigated for this purpose. Vectorcardiographic septal-to-basal ventricular peak activation time (SB-VPAT) was investigated in a preliminary pilot study. We hypothesize that SB-VPAT is a sensitive parameter for detection of systolic dysfunction due to preclinical DCM stage B2 and correlates with left ventricular size and function in Doberman Pinschers.

**Animals:**

One hundred and twenty-two Doberman Pinschers (98 control and 24 with systolic dysfunction due to preclinical DCM).

**Methods:**

Prospective cross-sectional study. All dogs underwent echocardiography, three-minute six or 12-lead ECG and RELF ECG. Based on echocardiographic evaluation, dogs were classified into a control group (including apparently healthy dogs and dogs with ventricular arrhythmia’s only) or a group with systolic dysfunction associated with preclinical DCM stage B2. ROC curves of SB-VPAT and its correlation with left ventricular size and function were analyzed.

**Results:**

SB-VPAT ≥33.5 ms had a sensitivity of 94.4% and specificity of 83.6% for the detection of systolic dysfunction due to preclinical DCM stage B2 (AUC 0.954, SD 0.022). Furthermore, SB-VPAT was strongly correlated with the left ventricular systolic diameter, systolic volume index and moderately inversely correlated with EF.

**Conclusions and clinical importance:**

In conclusion, SB-VPAT is a sensitive parameter to detect systolic dysfunction associated with preclinical DCM stage B2. Further investigation of its diagnostic potential compared to or in combination with other tools in a primary care veterinary setting is warranted.

## Introduction

Dilated cardiomyopathy (DCM) is the second most common acquired cardiac disease in dogs, with a cumulative prevalence of 58.8% in Doberman Pinschers (1). The preclinical stage is characterized by the presence of arrythmias (stage B1), echocardiographic changes (stage B2) (left ventricular systolic dysfunction and dilatation) or both and can last for several years before onset of the clinical stage (stage C) that is characterized by signs of congestive heart failure (1–5). Sudden cardiac death is reported in 25–30% of affected dogs (4, 5). Cardiac screening is important for breeding policy and prognosis as early diagnosis and appropriate treatment can increase life expectancy (6). Echocardiography and 24-h Holter electrocardiogram (ECG) monitoring are currently the gold standard for diagnosis of preclinical DCM in Dobermans. However, these tests are suboptimal for screening on a large scale as they are expensive and require a high level of expertise, limiting their availability in primary veterinary practice.

Cost-effective and readily available diagnostic tests in primary veterinary practice include N-terminal pro B-type natriuretic peptide (NT-proBNP) and cardiac troponin I (cTnI). A recent study showed a high sensitivity of 99.2% and low specificity 31.8% [area under curve (AUC) of 0.949] for NT-proBNP and cTnI combined to discriminate between Dobermans with and without echocardiographic signs of preclinical DCM [defined as LVIDs (mm) >0.1402 BW (kg) + 35.3 mm] (7). Potential drawbacks of these biomarkers are that they require blood sampling (with centrifugation and frozen transportation of plasma and serum), have breed dependent reference intervals, they display day-to-day variability and can be influenced by extra-cardiac diseases (4, 5, 8–11).

In humans, QRS prolongation on surface ECG is associated with ventricular chamber enlargement and dysfunction but lacks sensitivity as a diagnostic marker (9, 10). In dogs, the diagnostic utility of QRS duration on routine surface ECG for diagnosis of DCM is largely unknown (12, 13). Vectorcardiography (VCG) is a diagnostic technique that quantifies the magnitude and orientation of cardiac electrical forces over time, represented as a series of time-dependent vectors. By connecting the endpoints of these vectors, a vector loop is generated, capturing the spatial dynamics of electrical activity throughout the cardiac cycle. This three-dimensional representation of cardiac electrical forces provides a more comprehensive assessment compared to the one-dimensional data obtained from conventional electrocardiography (ECG) (14). The QRS loop on a VCG represents the sequential depolarization of the ventricles, with main vectors corresponding to septal depolarization (vector 1), left ventricular depolarization (vector 2) and basal region depolarization (vector 3) (15). The QRS duration on a surface ECG is determined by the alignment of all vectors during ventricular depolarization in relation to the recording leads. The onset, and more commonly the termination, of the QRS complex can be isoelectric in an individual lead, leading to vector cancellation, underestimation and variance of the actual QRS duration on a surface ECG (14, 16, 17).

Theoretically, vectorcardiographic measurement of the ventricular depolarization time could be more accurate due to its spatial resolution of the initial and terminal portion of the QRS (vector 1 and 3). In a small exploratory vectorcardiographic study involving 10 Doberman Pinschers (unpublished data), we identified a strong correlation between the end-systolic ventricular diameter and the duration (in milliseconds) between vector 1 and vector 3, which we named the septal-to-basal ventricular peak activation time (SB-VPAT). This parameter represents the time necessary from the maximum depolarization of the interventricular time to the maximum depolarization of the basal region, excluding early beginning of the interventricular depolarization and the late depolarization of the ventricular basal region.

The objective of this study is to prospectively evaluate the SB-VPAT parameter as a potential pre-screening biomarker for identifying Doberman Pinschers at elevated risk of systolic dysfunction associated with preclinical DCM stage B2. The study aims to assess the sensitivity and specificity of this parameter and to determine its correlation with left ventricular size and functional metrics.

## Materials and methods

### Animals

The study protocol was approved by the Ethical Committee of the Faculty of Veterinary Medicine and Bioscience Engineering at Ghent University (EC 2019-97). Only Doberman Pinschers were included due to the high prevalence of DCM in the breed and the homogeneity of the chest configuration. Client-owned Dobermans, presented for DCM screening or follow-up, were recruited prospectively at the faculty of veterinary medicine of Ghent University (Belgium) between September 2018 and May 2022 and the Ludwig Maximilians University in Munich (Germany) between October 2021 and May 2022. Eleven Dobermans were investigated off-site at a local event for Dobermans in Maastricht, The Netherlands.

Both apparently healthy and Dobermans affected with preclinical DCM (stage B1 and B2) were included in the study. Exclusion criteria were the presence of congestive heart failure, other heart diseases or significant systemic diseases, potentially influencing cardiac function (e.g., treatment with doxorubicin, hypothyroidism or critically ill). Thoracic radiographs were taken if there was a suspicion of congestive heart failure. To exclude taurine deficiency, blood taurine levels were determined in dogs receiving a non-traditional diet.

All participating Dobermans underwent a standard echocardiography, a three-minute 6- or 12-lead ECG and a vectorcardiography according to the RELF method modified from human medicine.

### Echocardiography and electrocardiogram

A standard echocardiogram was obtained by either a board-certified cardiologist or a resident under supervision of a diplomate, with particular attention to optimization of planes for left ventricular volume measurements. The echocardiography was conducted based on current general and Doberman specific guidelines for the diagnosis of canine DCM (3–5). The echocardiographic criteria for left ventricular dilatation or systolic dysfunction were: left ventricular internal diastolic diameter (LVIDd) >48 mm for a male and LVIDd >46 mm for a female dog, left ventricular internal systolic diameter (LVIDs) >36 mm, Simpsons method of discs (SMOD) for end-diastolic volume index (EDVI) >95 mL/m^2^, end-systolic volume index (ESVI) >55 mL/m^2^, and ejection fraction by Simpson’s method of discs (SMOD) <40%. Right parasternal long axis M-mode measurements were used to define the LVIDs and LVIDd. If the M-mode image was not reliable, short axis two-dimensional measurements were used. Additionally, LVIDdN and LVIDsN were calculated using Cornell normalization (18). All measurements were averaged for three cardiac cycles. A three minute 6- or 12-lead ECG was obtained at 50 mm/s.

Dobermans were assigned to the control group or preclinical DCM stage B2, based on echocardiographic criteria from the European Society of Veterinary Cardiology screening guidelines for dilated cardiomyopathy in Doberman Pinschers (4). Definitive diagnosis of a preclinical DCM phenotype was made if both left ventricular indexed volumes (mL/m^2^) were increased (ESVI and EDVI). Systolic dysfunction was diagnosed in cases where only ESVI was elevated while EDVI remained normal, provided the EF was also <40%, classifying these individuals within the preclinical DCM group. If there was a ventricular arrythmia on the ECG without significant abnormalities on the echocardiogram, the dog was allocated to the control group, which consequently included both apparently healthy and stage B1 Dobermans. It is not excluded that more dogs with preclinical DCM stage B1 are included in the control group, hence an 24-h Holter ECG in necessary for this diagnosis.

Treatment with medications such as pimobendan, atenolol, or sotalol was not considered an exclusion criterion. However, since the study aimed to evaluate the SB-VPAT parameter specifically in the context of systolic dysfunction associated with preclinical DCM, dogs receiving pimobendan prior to examination were excluded if their echocardiogram showed no abnormalities.

### Vectorcardiogram according to the modified RELF method

**Figure 1:**
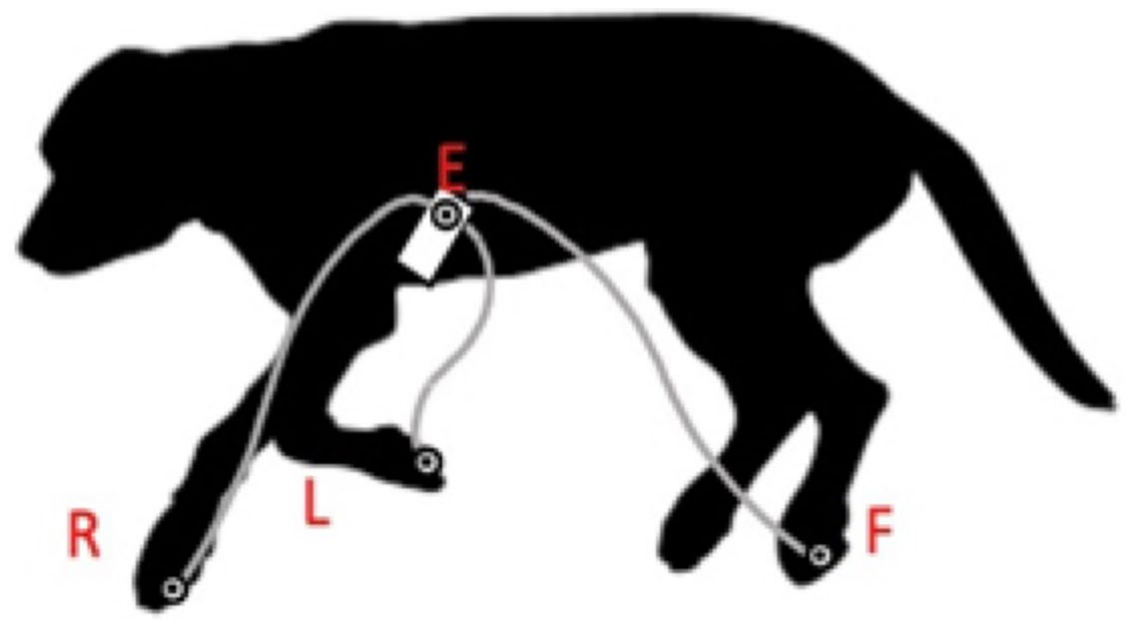
Electrode positioning for vectorcardiogram according to the modified human RELF method: dog in right lateral positioning. R: right front paw, E: located 2 cm to the left of the sternum at the 4–5th intercostal space, L: left front paw, and F: left hind paw.

**Figure 2:**
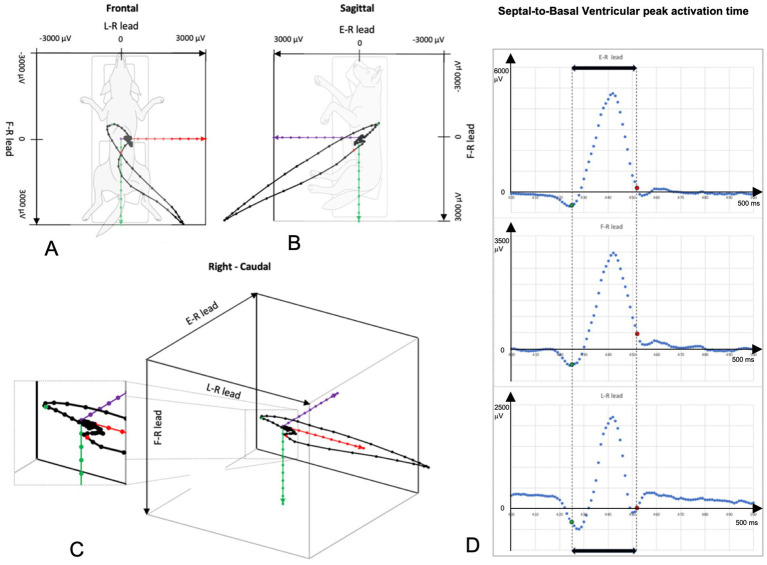
QRS loop in a three-dimensional orthogonal vectorcardiogram (VCG). Panels **A–C** show the frontal, sagittal and right-caudal views, respectively. Red axis is formed by the L-R lead, the green axis by the F-R lead and the purple axis the R-E lead. The green dot in panels **C,D** represents the maximal septal depolarization, (see also vector 1 in Figure 3). The red dot represents the maximal basal region depolarization (see also vector 3 in Figure 3). The septal-to-basal ventricular peak activation time (SB-VPAT) (bold black arrow) is the time between the green and red dot. Panel **D** depicts the individual RELF leads with the X-axis representing time in milliseconds and the Y-axis representing voltage in millivolts.

The RELF vectorcardiogram is a small prototype device consisting of one small panel board and four electrode wires. This device was chosen for its minimal lead placement requirements, demonstrating that VCG is a feasible and practical method for use in dogs. Placement of the electrodes was based on the RELF method for humans as described by El Haddad et al. (19) and modified for the dog (Figure 1). RELF is an acronym for the four electrode positions right (right front paw), exploratory (located 2 cm left of the sternum at the 4–5th intercostal space), left (left front paw), and foot (left hind paw). The three leads are generated by the voltage subtractions E-R, F-R, and L-R with one electrode in common (R). Voltages were measured simultaneously at sampling frequency of 1,000 Hz. The recorded leads provide a non-orthogonal three-dimensional vectorcardiogram (VCG). Three QRS complexes were extracted for each Doberman. VCG loops of the QRS complexes were created by plotting the three leads of the extracted QRS complex as coordinates in a three-dimensional orthogonal plot graph with L-R representing the X-axis, E-R the Y-axis, and F-R the Z-axis (Figure 2).

### Definition and calculation of septal-to-basal ventricular peak activation time

**Figure 3:**
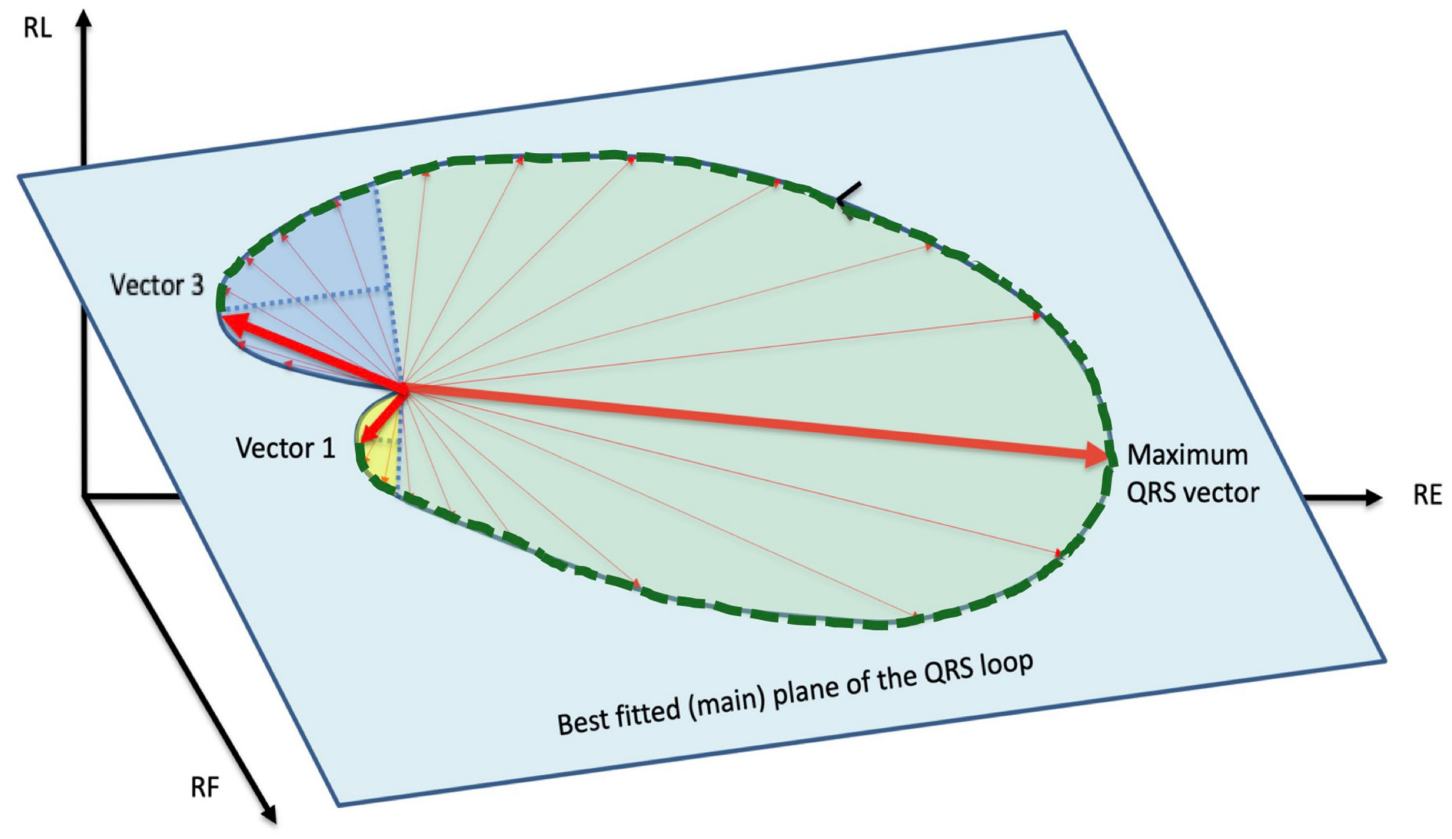
Septal-to-basal ventricular peak activation time (SB-VPAT): two best fitting parabolic planes are defined in the three-dimensional area of the QRS loop. The parabolic plane 1 (yellow) is the largest parabolic area fitting the depolarization phase of interventricular septum. Vector 1 is the depolarization vector at the vertex of this parabola. The parabolic area 2 (dark blue) is the maximum parabolic area fitting the depolarization phase of the basal regions. Vector 3 is the depolarization vector at the vertex of the parabola. The septal-to-basal ventricular peak activation time (SB-VPAT) is the time interval between vector 1 and vector 3, indicated with a dotted green line. The spatial orientations of best fitting parabolic planes can be different (out-of-plane) from the main plane of the QRS loop (green) (see Figure 2).

We define SB-VPAT on a QRS loop as the time interval from the maximum activation of the interventricular septum (ventricular depolarization vector 1) to the maximum activation of the basal regions of the ventricle (ventricular depolarization vector 3) according to the method illustrated in Figure 3. The QRS loop was considered unreliable and the measurement excluded if both maxima could not be easily identified.

### Statistical analysis

Data was analyzed using IBM SPSS Statistics Software Version 28. All variables were tested for normality with the Shapiro–Wilk test. Receiver-operating characteristic (ROC) curves were generated to calculate the area under the curve (AUC) for distinguishing dogs with systolic dysfunction due to preclinical DCM stage B2 from those without. Spearman correlation test was performed to determine the correlation of SB-VPAT with the echocardiographic measurements of left ventricular size and function (SMOD EDVI and ESVI, LVIDd, LVIDs, LVIDdN, LVIDsN, EF). Inter- and intraobserver variability was obtained with intraclass correlation coefficient (ICC) and coefficients of variations (CV) respectively. To evaluate the inter- and intra-observer variability, VCG’s of 10 Dobermans (five with and five without DCM) were blindly measured by MG and another, unexperienced observer. All other VCG’s were measured by one observer (MG).

## Results

A total of 122 Doberman Pinschers were included in the study, four Dobermans were excluded from the study due to normal echocardiographic parameters under treatment with pimobendan.

The control group consisted of 98 Dobermans with mean bodyweight of 36.28 kg (±6.03) and mean age of 5.66 years (±2.56). Seven of the Dobermans in the control group had ventricular premature complexes (VPC’s) during the three-minute ECG recording of which three were treated with sotalol and three with amiodarone at the point of examination. The preclinical DCM stage B2 group included 24 Dobermans with mean bodyweight of 35.81 kg (±6.37); and mean age of 6.77 years (±2.27). In this group, five Dobermans had only systolic dysfunction without ventricular dilation and were not receiving pimobendan at the time of examination. The other Dobermans in the preclinical DCM stage B2 group, with systolic dysfunction and ventricular dilation, were receiving pimobendan at the time of examination except for one, with four dogs treated with sotalol and one dog receiving amiodarone at the time of examination. A RELF VCG was registered in all dogs (*n* = 122) and 79 recordings were of sufficient quality for analysis, more specifically 61/98 (62.2%) of the control group and 18/24 (75.0%) of the preclinical DCM stage B2 group. The echocardiographic parameters and SB-VPAT values are summarized in Table 1.

**Table 1:** Descriptive statistics (mean and standard deviation) of all echocardiographic parameters in the control and preclinical dilated cardiomyopathy group, septal-to-basal ventricular peak activation time (SB-VPAT).

Parameter	Control (*n* = 61)	Preclinical stage B2 (*n* = 18)
SB-VPAT (ms)	31.09 ± 2.98	37.77 ± 2.64
LVIDd (mm)	40.54 ± 3.49	48.57 ± 5.17
LVIDdN	1.32 (1.11–1.53)	1.57 ± 0.16
LVIDs (mm)	30.06 ± 3.59	40.82 ± 5.41
LVIDsN	1.02 (0.92–1.12)	1.35 ± 0.16
EDVI (mL/m^2^)	76.08 ± 11.98	107.33 ± 20.05
ESVI (mL/m^2^)	38.73 ± 8.96	64.11 (53.27–74.95)
%EF	49.35 ± 8.49	34.84 ± 9.27

LVIDd, left ventricular internal diastolic diameter; LVIDdN, left ventricle diameter in diastole and the normalized; LVIDs, left ventricular internal systolic diameter; LVIDdN, left ventricular internal diastolic diameter left ventricle diameter in systole and the normalized; EDVI, end diastolic volume index; ESVI, end systolic volume index; EF, ejection fraction. LVIDdN, LVIDsN in the control group and ESVI in the preclinical group were not normally distributed (Shapiro–Wilk test *p* < 0.05) and described as median with interquartile range.

Intraclass correlation coefficient indicated an excellent inter-observer agreement for measurement of SB-VPAT between the two observers (ICC 0.97). The intra-observer coefficients of variation (CV) for measurement of SB-VPAT were 8.19 and 7.38%, suggesting that each observer demonstrated good consistency when measuring SB-VPAT, with only minor variation in their repeated measurements.

**Figure 4:**
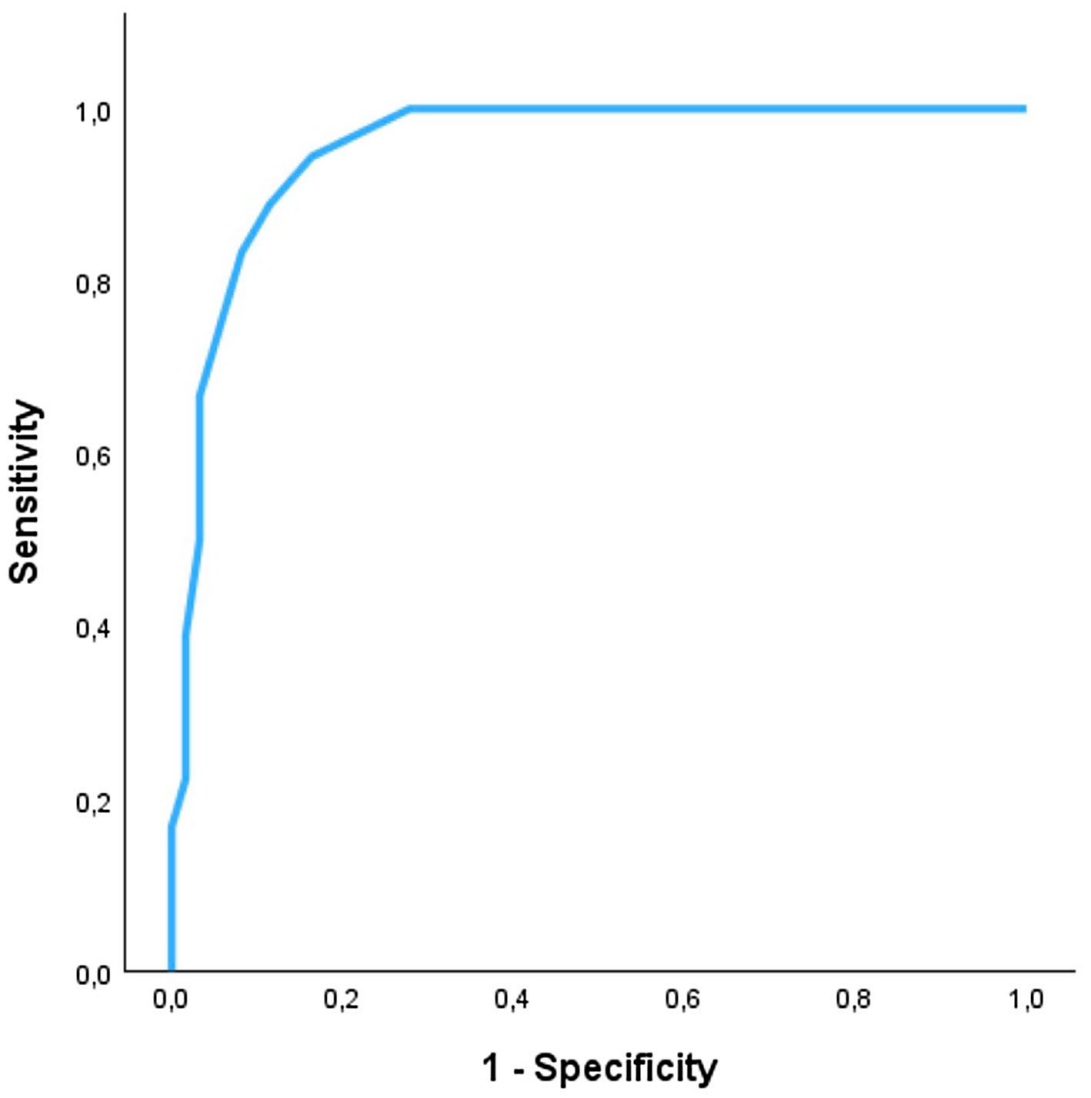
The accuracy of septal-to-basal ventricular peak activation time (SB-VPAT) to detect preclinical dilated cardiomyopathy stage B2 vs. control in Dobermans. Receiver operating characteristics curve (ROC) of the 18 recordings in Dobermans with preclinical dilated cardiomyopathy and 61 recordings in healthy Dobermans. ROC area is 0.954 (95% CI 0.910–0.997, SD 0.022).

**Figure 5:**
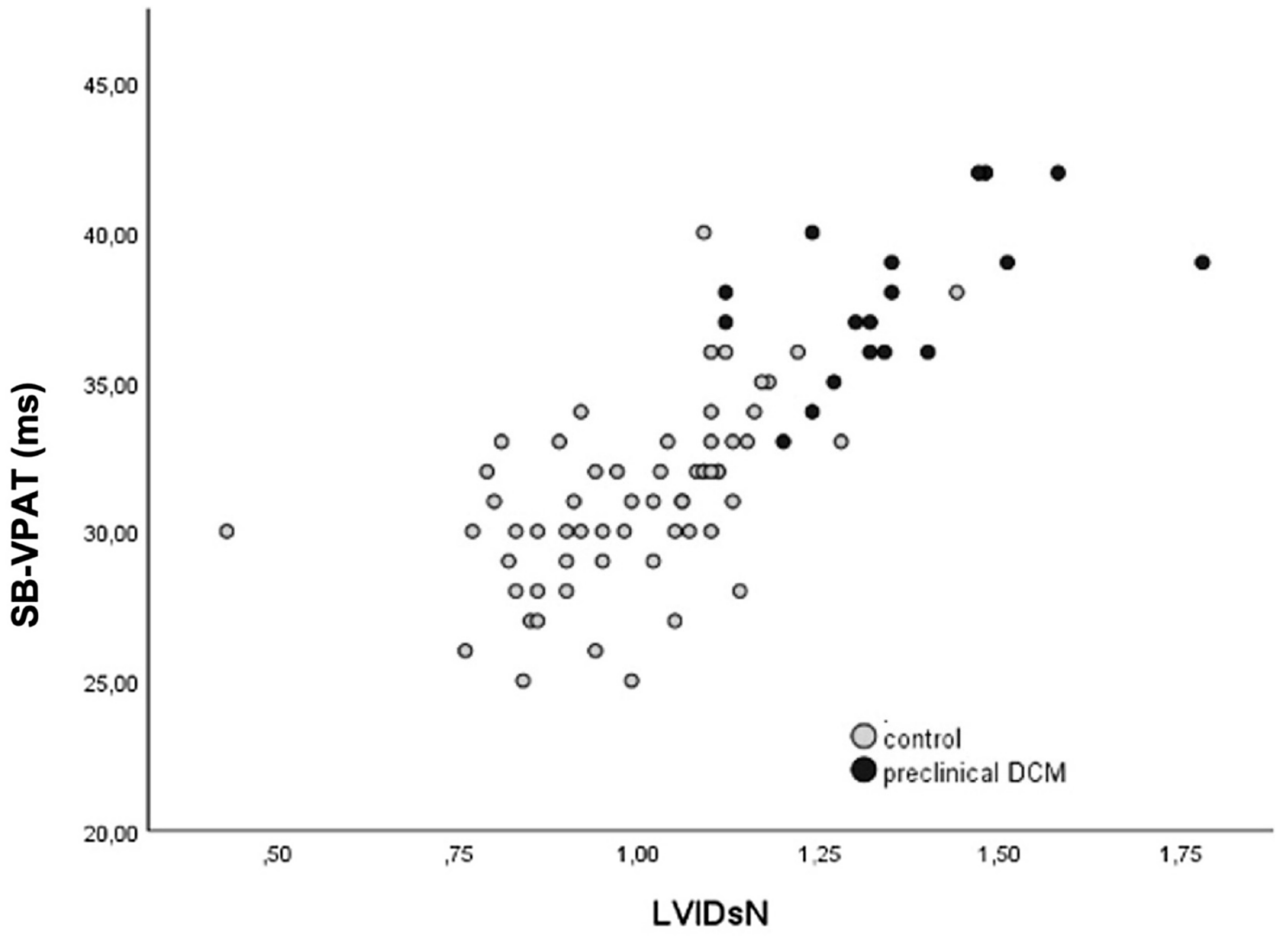
Correlation of septal-to-basal ventricular peak activation time (SB-VPAT) and the normalized left ventricular diameter in systole (LVIDsN) in control and the preclinical dilated cardiomyopathy group stage B2 (*r* = 0.78, *p* < 0.001).

For the detection of systolic dysfunction due to preclinical DCM stage B2, ROC analysis of SB-VPAT indicated a sensitivity of 94.4% and a specificity of 83.6% when using a cut-off of 33.5 ms (AUC 0.954, 95% CI 0.910–0.997, SD 0.022) (Figure 4). Furthermore, SB-VPAT is strongly correlated with LVIDsN (*r* = 0.78, *p* < 0.001), ESVI (*r* = 0.71, p < 0.001) and moderately inversely correlated with EF (*r* = 0.65, p < 0.001) (Table 2 and Figure 5).

**Table 2:** Correlation of septal-to-basal ventricular peak activation time (SB-VPAT) with the echocardiographic parameters.

	LVIDd	LVIDs	LVIDdN	LVIDsN	EDVI	ESVI	EF
Coefficient	0.70	0.75	0.69	0.78	0.54	0.71	−0.65
Sig. (one-tailed)	<0.001	<0.001	<0.001	<0.001	<0.001	<0.001	<0.001
*N*	79	79	78	78	79	79	79

LVIDd, left ventricle diameter in diastole; LVIDs, left ventricle diameter in systole; LVIDdN, left normalized ventricle diameter in diameter by Cornell; LVIDsN, left normalized ventricle diameter in systole by Cornell; EDVI, end diastolic volume indexed; ESVI, end systolic volume indexed; EF, ejection fraction.

## Discussion

This study illustrates that septal-to-basal ventricular peak activation time (SB-VPAT), a novel vectorcardiographic parameter derived from the ventricular depolarization time, can distinguish apparently healthy and DCM stage B1 Doberman Pinschers from dogs with systolic dysfunction due to preclinical DCM stage B2 with high sensitivity (94.4%, AUC 0.954) at a cutoff value of 33.5 ms. Furthermore, SB-VPAT correlated with echocardiographic measurements of left ventricular size and function.

In this study, the RELF vectorcardiographic device, validated in humans for detection of ST-segment changes, was adapted for use in dogs. With this RELF device, a method was created to construct a vector loop from which ventricular depolarization time can be derived. Identification of the beginning and ending of the depolarization time on ECG or on the vector loop is considered unprecise because noise interferes with the direction and size of the initial and terminal low-voltage vectors. The noise interference decreases as higher voltages are reached during the depolarization time, therefore the loop between vector 1 and 3 is least influenced by noise. The time between vector 1 and vector 3 represents the largest and middle part of the systolic depolarization time, corresponding with the main biventricular activation times (free wall activation of both left and right ventricle, which is affected in diseased dogs with DCM). This Septal-to-Basal Ventricular peak activation time (SB-VPAT) may therefore represent the activation time of the ventricles more accurately and precisely. Compared to total QRS duration, the initial and latest activation times of the septal and basal segments, respectively, are not included with the SB-VPAT method. These parts of the septal and basal activations contribute to the global QRS duration, but the contribution of these segments to the overall ventricular activation is limited and thus may have little added value to discriminate normal from systolic dysfunction of the heart.

Looking at the relationship between SB-VPAT and echocardiographic measurements, SB-VPAT is correlated more strongly with left ventricular systolic parameters than with left ventricular diastolic parameters. This could be explained by the indirect correlation between conduction delay and systolic dysfunction. If SB-VPAT reflects a conduction delay associated with pathophysiological changes seen in DCM, it is biologically plausible that the correlation is highest with the parameters for systolic function.

In DCM, myofiber degeneration and replacement by collagen and adipocytes are caused by multiple pathophysiological processes, such as impaired oxidative production of ATP and reduction of myoglobin concentrations, resulting in decreased or biphasic contractility, subsequent systolic dysfunction, volume overload and eccentric hypertrophy (2). The duration of ventricular depolarization can be increased due to eccentric hypertrophy, but this cannot be distinguished from prolonged depolarization secondary to a ventricular conduction delay (bundle branch block, ventricular desynchrony) (13). An incomplete bundle branch block may be present in dogs with DCM due to degeneration of cardiac myocytes and was not excluded in this study due to the absence of a full 12-lead ECG of every dog. In case of complete bundle block, QRS duration on surface ECG exceeds a duration of 80 ms, which was the case in three Dobermans. A bundle branch block could increase the SB-VPAT parameter. From a clinical perspective, an increased SB-VPAT could indicate an enlarged left ventricle or a conduction delay, both indicating ventricular pathology in Dobermans.

Additionally, anti-arrhythmic drugs such as sotalol and amiodarone, can also increase QRS and QT interval duration (20, 21). Some dogs in both the preclinical DCM group and the control group were treated with these medications, with five of 18 dogs in the preclinical DCM stage B2 group (one treated with amiodarone and four with sotalol) and six of 61 dogs in the control group (three treated with amiodarone and three with sotalol). Amiodarone, a class III anti-arrhythmic agent, also possesses sodium- and calcium-channel blocking properties, which can lead to a prolongation of the QRS duration (21). In contrast, sotalol primarily exerts its effects through potassium-channel blockade, with minimal influence on QRS duration and more on QT interval. Consequently, amiodarone may have a greater impact on QRS duration compared to sotalol. While it cannot be ruled out that these drugs may have influenced QRS duration, the degree of their influence remains unknown. Moreover, the sample size of treated dogs is too small to determine whether there is a significant difference between those treated with anti-arrhythmic drugs and those who were not, precluding definitive conclusions.

When the correlation of SB-VPAT with LVIDsN is visualized in a scatter plot, we recognize that the correlation becomes less strong at the upper range of the diameters (Figure 5). This may be explained by the fact that cardiac enlargement will not be linear to the conduction delay and is anatomically limited in size. The potential clinical purpose of the SB-VPAT parameter as a pre-screening test is to detect subtle changes between a normal heart versus systolic dysfunction associated with preclinical DCM. Most severely enlarged ventricles are found in dogs with clinical DCM, not the target population for screening.

For the detection of systolic dysfunction due to preclinical Doberman DCM in stage B2, the RELF device may be a sensitive, cost-effective and accessible pre-screening method. Compared to cardiac biomarkers, SB-VPAT is immediately available, cost-effective and non-invasive, since it only requires placement of four electrodes on convenient body locations for a short time. The recording can also display valuable information concerning ventricular arrhythmias. One study investigated the combined value of biomarkers and physical examination for the detection of preclinical DCM in Dobermans (7). The ROC (AUC 0.949) of the combined biomarkers, NT-proBNP and cTnI, seems ideal for pre-screening, but the study population is not fully comparable to ours due to a different group assignment. The study used preclinical DCM as the target group but used a different control group including Dobermans with equivocal DCM (ventricular dilation without systolic dysfunction), Dobermans with ECG abnormalities or with mitral valve disease. Despite the high AUC of the multiparametric approach of physical examination and cardiac biomarkers, there are disadvantages such as management of the blood analysis (centrifuge, frozen shipment), venipuncture and no “bedside” result.

Of interest, the time points of the SB-VPAT parameter marked on the three separate RELF ECG’s (created by E-R, L-R and F-R lead) were not consistently located at an identifiable point of the surface ECG (Figure 2). Theoretically, these time points are comparable with the Q and S wave on the surface ECG, but the identification of these points is highly depended of the lead and noise interference. This highlights the usefulness of a three-dimensional measurement using VCG, creating more reliable reference points on the VCG.

A limitation of this study is the absence of a significant number of Dobermans in stage B1 not treated with sotalol or amiodarone, i.e., preclinical DCM without echocardiographic detectable changes but with ventricular arrhythmias. Further research is interesting to evaluate the sensitivity of the SB-VPAT in this population of stage B1, especially since it is still unknown if prolongation of the ventricular depolarization is caused by the anatomic enlargement of the ventricle or due to conduction disturbances caused by the degeneration of ventricular myocytes. Currently, it is unknown if Dobermans with a significant amount of VPC’s on 24 h Holter ECG show changes of the SB-VPAT parameter. Furthermore, as described above, no sub analysis was performed to investigate drug effects from sotalol, amiodarone or pimobendan. Anti-arrhythmic drugs may affect QRS duration (probably more amiodarone then sotalol) and pimobendan affects ventricular size (20, 21). Although this may have influenced the IVB-peak interval parameter in some of the included dogs, the impact on the results is considered minimal, a potential drug effect is balanced since a similarly low number of dogs were treated with anti-arrhythmic in both groups. Only one dog in the preclinical DCM group and three in the control group were treated with amiodarone, which could potentially have affected the ROC analysis negatively, meaning that the true sensitivity might be higher. Furthermore, dogs treated with pimobendan who had normal echocardiographic parameters were excluded from the analysis.

Another limitation of this study was the high number of RELF measurements of low quality, resulting in only 79 of the 122 RELF recordings that were qualified for analysis. The significant amount of noise in many recordings could be explained by to the low contact stability between the paws and the electrode patch due to the irregular surface of the paw or due to calluses. Muscle contractions due to stress or muscle tension can be another explanation of the electrical disturbance on the RELF ECG. After recognizing this problem, we also obtained RELF measurements using alligator clips on the elbows and knee of the Doberman. As described in literature, there is no voltage difference between the proximal and distal part of the limbs in a dog (13). This significantly increased the quality of the RELF ECG’s without changing the morphology of the RELF VCG. These easy adaptations could make this method more accessible in veterinary practices. In this study, we only investigated one type of thorax configuration. Mesomorphic or brachymorphic breeds might have different morphology of the RELF VCG, causing difficulties in identifying vector 1 and 3, so further investigation is needed before this parameter can be applied to other dog breeds. To calculate the SB-VPAT we used software that is currently not commercially available in veterinary practices. To further implement this method into practice, an algorithm is required to calculate the SB-VPAT automatically. Future longitudinal studies including healthy, equivocally affected and DCM stage B1 Dobermans are necessary to further investigate the performance of IVB-peak interval as a pre-screening parameter and compare it to cardiac biomarkers.

In conclusion, this study illustrated the utility of the SB-VPAT parameter as a reliable indicator of systolic dysfunction associated with preclinical DCM in Doberman Pinschers. The parameter demonstrated high sensitivity for detecting systolic dysfunction and showed a strong correlation with echocardiographic measures of left ventricular systolic function. Further research is recommended to explore its diagnostic value, either as a standalone tool or in combination with other screening methods, within primary care veterinary practice.

## Data Availability

The raw data supporting the conclusions of this article will be made available by the authors, without undue reservation.
